# Urine Stasis Predisposes to Urinary Tract Infection by an Opportunistic Uropathogen in the *Megabladder* (*Mgb*) Mouse

**DOI:** 10.1371/journal.pone.0139077

**Published:** 2015-09-24

**Authors:** Brian Becknell, Ahmad Z. Mohamed, Birong Li, Michael E. Wilhide, Susan E. Ingraham

**Affiliations:** 1 Nephrology Section, Nationwide Children’s Hospital, Columbus, OH, United States of America; 2 Department of Pediatrics, Ohio State University School of Medicine, Columbus, OH, United States of America; 3 Center for Clinical and Translational Research, The Research Institute at Nationwide Children’s, Columbus, OH, United States of America; 4 Department of Urology, University of Louisville, Louisville, KY, United States of America; Emory University, UNITED STATES

## Abstract

**Purpose:**

Urinary stasis is a risk factor for recurrent urinary tract infection (UTI). Homozygous mutant *Megabladder* (*Mgb*-/-) mice exhibit incomplete bladder emptying as a consequence of congenital detrusor aplasia. We hypothesize that this predisposes *Mgb*-/- mice to spontaneous and experimental UTI.

**Methods:**

*Mgb-/-*, *Mgb+/-*, and wild-type female mice underwent serial ultrasound and urine cultures at 4, 6, and 8 weeks to detect spontaneous UTI. Urine bacterial isolates were analyzed by Gram stain and speciated. Bladder stones were analyzed by x-ray diffractometry. Bladders and kidneys were subject to histologic analysis. The pathogenicity of coagulase-negative *Staphylococcus* (CONS) isolated from *Mgb-/-* urine was tested by transurethral administration to culture-negative *Mgb-/-* or wild-type animals. The contribution of urinary stasis to CONS susceptibility was evaluated by cutaneous vesicostomy in *Mgb-/-* mice.

**Results:**

*Mgb*-/- mice develop spontaneous bacteriuria (42%) and struvite bladder stones (31%) by 8 weeks, findings absent in *Mgb*+/- and wild-type controls. CONS was cultured as a solitary isolate from *Mgb-/-* bladder stones. Bladders and kidneys from mice with struvite stones exhibit mucosal injury, inflammation, and fibrosis. These pathologic features of cystitis and pyelonephritis are replicated by transurethral inoculation of CONS in culture-negative *Mgb-/-* females, whereas wild-type animals are less susceptible to CONS colonization and organ injury. Cutaneous vesicostomy prior to CONS inoculation significantly reduces the quantity of CONS recovered from *Mgb*-/- urine, bladders, and kidneys.

**Conclusions:**

CONS is an opportunistic uropathogen in the setting of urinary stasis, leading to enhanced UTI incidence and severity in *Mgb-/-* mice.

## Introduction

Congenital obstructive uropathy (COU) ranks among the most common causes of pediatric chronic kidney disease (CKD) and end stage renal disease (ESRD).[1] Recurrent urinary tract infections (UTIs) are common in patients with COU, including posterior urethral valves (PUV) and prune belly syndrome. UTI prolongs hospitalization in patients with COU, resulting in 30% increase in hospital costs.[2] UTI severity increases in the setting of urinary tract obstruction, placing patients at risk for severe pyelonephritis and urosepsis. Pyelonephritis and urosepsis are risk factors for CKD progression in COU patients with PUV and prune belly syndrome.[3,4,5] These observations justify efforts to aggressively prevent, detect, and treat UTIs in COU patients. However, the lack of animal models of spontaneous UTI has limited research in this arena.

The *Megabladder* (*Mgb*) mouse is a unique genetic model of COU.[6,7] *Mgb* arose as a consequence of a complex genomic rearrangement during transgenesis. Whereas mice with a single *Mgb* allele (*Mgb+/-*) are phenotypically normal, *Mgb-/-* embryos exhibit impaired mesenchymal compaction during detrusor muscle development. [7,8] Detrusor agenesis results in poor contractility and impaired urine outflow. The *Mgb-/-* mouse has been previously characterized as a model of a congenital functional obstruction at the level of the bladder, leading to hydroureteronephrosis and renal injury beginning *in utero*.[1,6,7,8] The atonic *Mgb*-/- bladder parallels that seen in children with prune belly syndrome, Hinman syndrome, and certain cases of myelomeningocele. This contrasts with detrusor hypertrophy associated with anatomic bladder obstruction, such as PUV.[1]

*Mgb*-/- males with severe hydroureteronephrosis require urinary diversion by cutaneous vesicostomy (CV) to prevent death by 5–6 weeks.[6,9] Bladder stones and UTI can be induced in up to 30% of *Mgb*-/- males by CV with polypropylene suture.[10,11,12] Compared to their male counterparts, *Mgb*-/- females exhibit reduced severity of hydronephrosis, prolonged lifespan, decreased incidence of ESRD, and rarely require surgical rescue. Hence, female *Mgb-/-* mice can serve as a model of urinary stasis and long-term CKD secondary to a functional obstruction. In routine care of *Mgb-/-* animals, we identified UTI associated with struvite urolithiasis in moribund females, none of whom had undergone surgical intervention (unpublished observations). We undertook this study to test the hypothesis that urine stasis predisposes to spontaneous UTI in *Mgb*-/- females.

## Methods

### Study approval

Use of the *Mgb*-/- mouse and experimental UTI models was approved by The Research Institute at Nationwide Children’s Hospital Institutional Laboratory Animal Care and Use Committee (Welfare Assurance Number A3544-01), protocols AR13-00057 (BB) and AR13-00001 (SEI).

### *Mgb-/-* model

*Mgb*-/- mice are maintained on a pure FVB/N genetic background. Throughout this manuscript, “wild-type” (WT) refers to the FVB/N parental strain. All mice were maintained in a transgenic barrier facility on a 12-hour light-dark cycle and fed standard chow (Harlan Laboratories, Indianapolis, IN).

### Microbiology

Urinary tract organs were sterilely dissected from euthanized mice and homogenized in phosphate-buffered saline. Organ and stone homogenates were serially plated on Luria Broth (LB) agar without antibiotic selection. Colonies were speciated (ChildLab, Nationwide Children’s Hospital) and frozen as glycerol stocks. CONS292 is a urease(+) CONS strain recovered from the bladder stone of a spontaneously infected *Mgb*-/- mouse.

### Experimental UTI model

6-week-old FVB/N female mice were purchased (Jackson Laboratories, Bar Harbor, ME) and rested for 1 week. Immediately prior to introduction of inoculum, urine was tested for pH and absence of bacteria. One day prior to inoculation, CONS292 was seeded into LB medium and cultured statically at 37°C for 16 hours. On the day of inoculation, bacteria were harvested by centrifugation and resuspended in phosphate buffered saline (PBS). Under isoflurane anesthesia, mice underwent urethral catheterization.[13] 10^8^ colony-forming units (CFU) of CONS292 were transurethrally introduced in 50 μl saline. The inoculum was confirmed by plating on LB agar. Urine was collected from the urethral meatus at the specified intervals following inoculation. 14 days post inoculation (dpi), animals were re-anesthetized for ultrasound, urine collection, and then euthanized. Bladders and kidney homogenates were plated on LB agar to enumerate bacterial burden.

### Ultrasound

Anesthetized *Mgb*-/- mice underwent ultrasonography at 4, 6, and 8 weeks of age.[6,14] All animals undergoing experimental UTI had ultrasound prior to inoculation and again 14 dpi. Although we observed excellent agreement between ultrasound and necropsy observation of stones, ultrasound was used as a screening modality to determine if the stone burden endpoint criterion for euthanasia was reached at early time points. Necropsy was considered definitive for the diagnosis of stones.

### Urine and stone evaluation

Urine was obtained from the urethral meatus (WT or *Mgb+/-* animals) or by direct aspiration from the bladder (*Mgb-/-*). Urine pH was determined by indicator strips (pH range 4.5–10, Cardinal Health, Waukegan, IL). Urine was centrifuged at 3000 x *g* for 5 minutes, and the sediment was evaluated by light microscopy using a DMI4000 microscope equipped with DFC450C camera (Leica Microsystems, Wetzler, Germany). Bladder stone composition was evaluated by x-ray diffractometry (Louis C. Herring and Company, Orlando, FL).

### Histologic analysis

Dissected tissues were fixed in 4% paraformaldehyde for 24 hours, transferred to 70% ethanol, and paraffin embedded. Four-micron-thick sections were evaluated by hematoxylin and eosin (H&E). Images were captured using the BZ-9000 microscope (KEYENCE, Osaka, Japan) or DMI4000 microscope equipped with DFC450C camera (Leica Microsystems, Wetzler, Germany).

### Data analysis

Based on our initial observations, we determined that a cohort of 17 animals would provide >80% power to detect spontaneous urolithiasis and/or bacteriuria at a frequency of 5% and 90% power to detect an incidence of 10% during the study period. To allow for animal loss or other unforeseen circumstances, we established a cohort of 19 consecutive *mgb*-/- female mice. Due to the small sample size, Fisher’s exact test was used for the analysis of categorical data. To determine significance of quantitative data, Mann-Whitney U test was used. Except as noted, *p* < 0.05 was considered statistically significant. All statistical tests were performed using Stata v.11.1 (StataCorp, College Station TX).

## Results

### Coagulase-negative *Staphylococcus* species are associated with struvite urolithiasis and urinary tract colonization in *Mgb-/-* females

**Fig 1:**
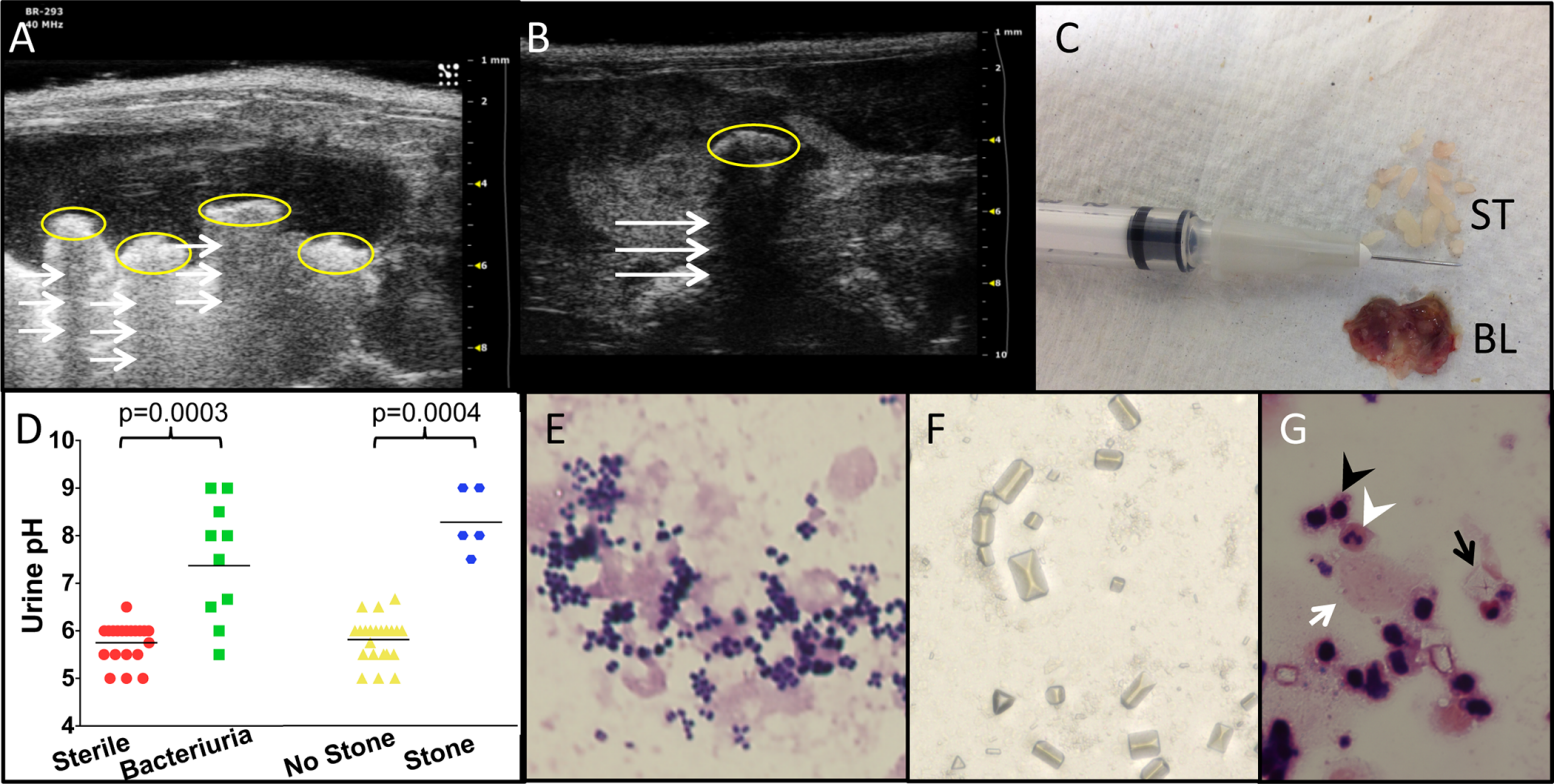
*Mgb*-/- female mice exhibit struvite urolithiasis associated with spontaneous UTI. Ultrasound of *Mgb*-/- female mice demonstrates bladder (**A**) and kidney (**B**) echogenic foci representing stones (circled) with acoustic shadowing (arrows). (**C**) Bladder stones (ST) harvested from a *Mgb*-/- female bladder (BL) with a spontaneous UTI. Stone analysis confirmed struvite (magnesium ammonium phosphate) as the major chemical component. (**D**) Urine from *Mgb*-/- females with bladder stones or UTI is alkaline compared to *Mgb*-/- females with sterile urine or lacking stones. *p* values by Mann-Whitney U test are indicated above the graph. (**E**) Gram stain of urine sediment from *Mgb*-/- females with struvite bladder stones demonstrates Gram-positive cocci in clusters consistent with *Staphylococcal* species. 100x objective. (**F**) Bright field light micrograph of urine sediment from *Mgb*-/- females with struvite bladder stones demonstrates typical “coffin-lid” appearance of struvite crystals. (**G**) Wright-Giemsa stain showing struvite crystals (black arrow), mononuclear and polymorphonuclear leukocytes (black and white arrowheads, respectively), and desquamated urothelial cells (white arrow) in urine sediment from an *Mgb*-/- female with spontaneous bacteriuria. 100x objective.

To investigate the incidence and etiology of bladder stones in *Mgb*-/- females, we performed serial ultrasounds and urine cultures in a cohort of 19 consecutive 4 week old *Mgb*-/- female mice. If stones were absent and urine remained sterile, we repeated ultrasound and cultures at 6 and 8 weeks of age. At 8 weeks, all animals were euthanized and tissues were collected for culture and histopathology. We identified bladder stones in 6/19 *Mgb*-/- mice (31.6%; Fig 1A). In two instances, we observed concurrent kidney and bladder stones (Fig 1B). At necropsy, we enucleated multiple stones from dissected *Mgb*-/- bladders (Fig 1C). All bladder stones were composed of ≥ 90% struvite with up to 10% apatite, consistent with an infectious origin.

We identified bacteriuria in 8/19 (42%) of *Mgb-/-* mice. Urine cultures from six animals with bladder stones grew coagulase negative *Staphylococcus* (CONS) non-*saprophyticus* species as solitary isolates. Urine from two animals lacking bladder stones contained *Enterococcus* species as solitary isolates. Cultures from homogenized stones grew coagulase negative *Staphylococcus* (CONS) non-*saprophyticus* species as a solitary isolate in all six urolithiasis cases.

We confirmed that CONS from *Mgb-/-* stones expresses urease, the enzyme responsible for hydrolyzing urea to ammonia, a required step in infection stone formation.[15] As expected in urine colonized by urea-splitting organisms, urine pH was significantly more alkaline in *Mgb*-/- females with stones (*p* = 0.0003, Mann-Whitney U test) or bacteriuria (p = 0.0004), compared to *Mgb*-/- females with sterile urine and absent stones (Fig 1D). Urinary sediment contained Gram-positive cocci in clusters (Fig 1E), as well as struvite crystals (Fig 1F and 1G) associated with polymorphonuclear leukocytes and urothelial cells (Fig 1G).

**Fig 2:**
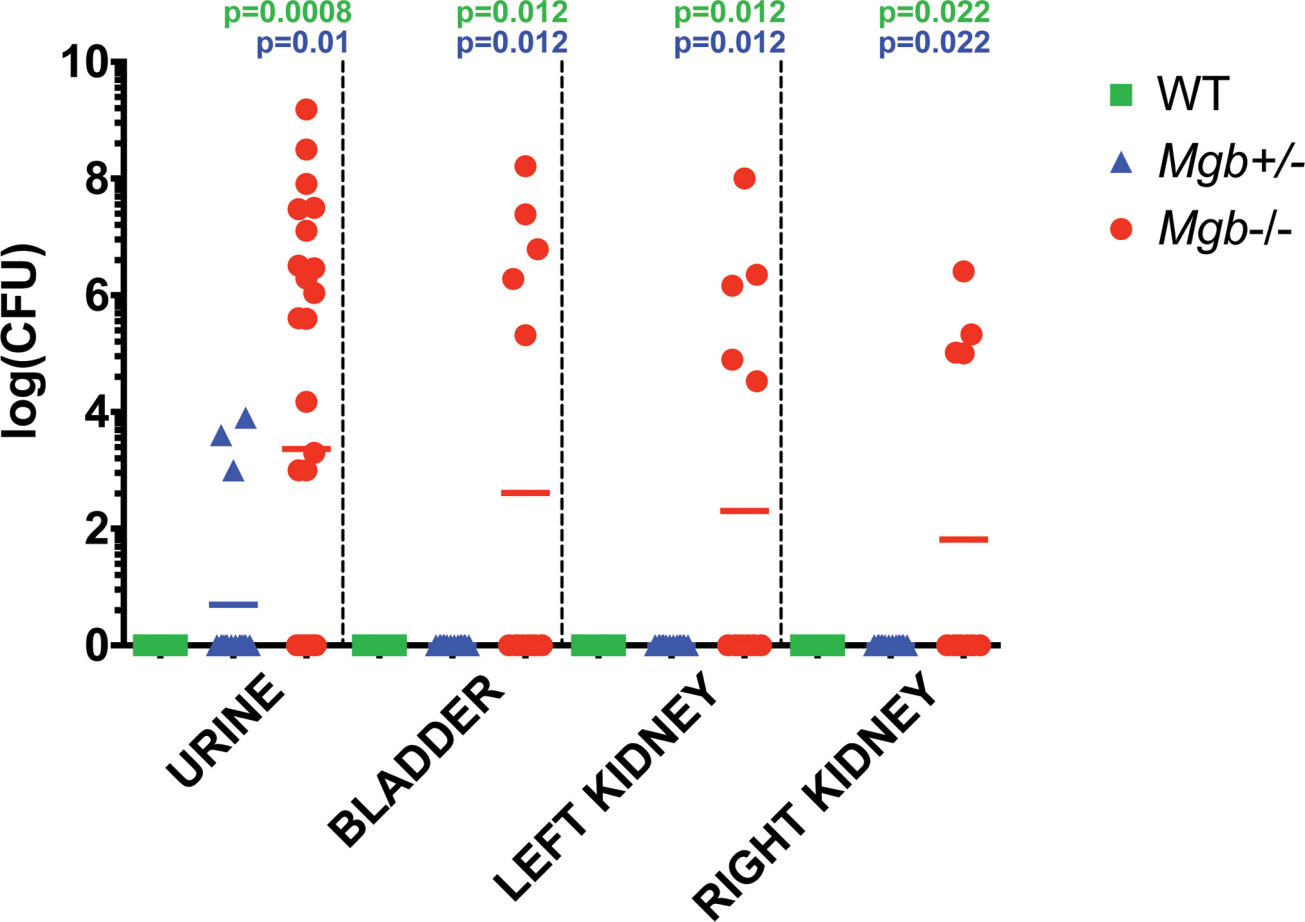
Spontaneous bacterial colonization by CONS in *Mgb-/-* females versus *Mgb+/-* and WT controls. Bacteria recovered from urine (CFU/ml), bladders, and kidneys (CFU/organ) were enumerated following serial dilution and culture under non-selective conditions. CONS was identified using standard microbial techniques. *p* values by Mann-Whitney U test are indicated above the graph. Bacterial burden was significantly higher in *Mgb-/-* compared to WT (green text) and *Mgb+/-* (blue text).

By eight weeks of age, urine from 8/19 (42.1%) Mgb-/- females contained > 100,000 colony forming units (cfu) bacteria/ml, identified as CONS in hosts with alkaline urine or a bladder stone. Animals with bladder stones also displayed evidence of lower and upper urinary tract colonization, based on recovery of CONS from bladder and kidney homogenates (Fig 2). Rare positive urine cultures from *Mgb-/-* females with acidic acid and absence of bladder stones grew *Escherichia coli* or *Enterococcus* species. We analyzed a cohort of consecutive *Mgb+/-* and wild-type (WT) mice from the parental FVB/N strain, to determine if the incidence and magnitude of urinary tract colonization were distinct from *Mgb-/-* animals. Whereas WT urine was consistently sterile, we detected low levels of spontaneous bacteriuria (1000–8000 cfu/ml) in 3/14 urine samples from *Mgb+/-* mice. The magnitude of CONS recovered from the urinary tract of *Mgb-/-* mice was significantly higher than that observed in the *Mgb+/-* and WT cohorts (Fig 2). We observed no alkaline urine or bladder stones in *Mgb+/-* or WT mice (Fisher exact test, *p* = 0.027).

### Cystitis and Pyelonephritis in *Mgb-/-* mice

**Fig 3:**
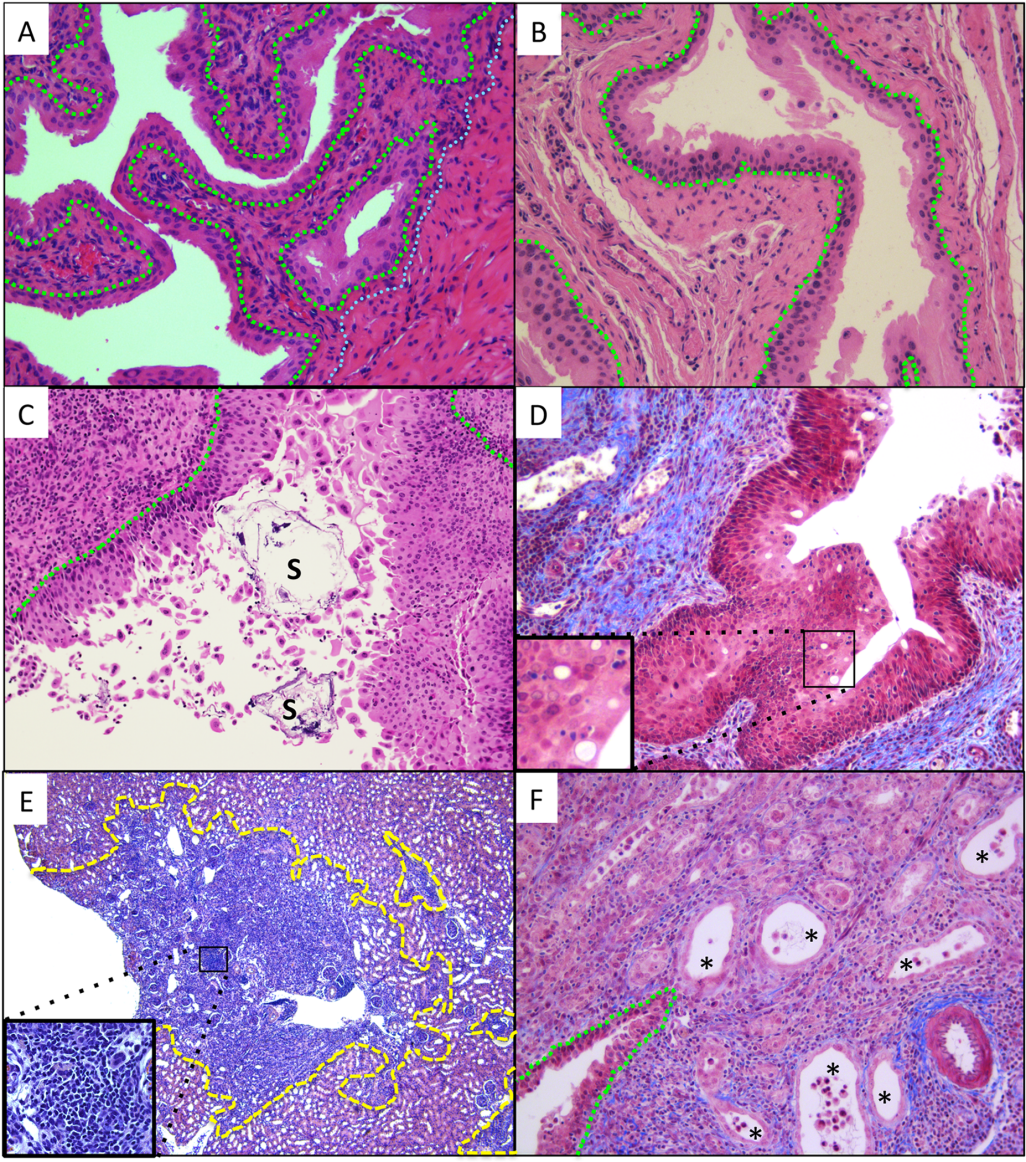
Histopathology of *Mgb*-/- bladders and kidneys with spontaneous infection and struvite stones. **(A)** WT bladders exhibit a multilayered but compact urothelium over the connective tissue of the lamina propria, and thick bundles of smooth muscle comprising the muscularis propria. Green dashed line demarcates the urothelial basement membrane. White dotted line indicates the boundary between submucosa and muscularis propria. H&E, 20x objective. **(B)** Uninfected *Mgb*-/- bladders demonstrate a thickened urothelium and submucosa, and lack the muscularis propria due to the *Mgb* mutation. Detached urothelium as well as other cellular and non-specific debris are found in the bladder lumen. H&E, 20x objective. **(C)** *Mgb*-/- bladders exhibit luminal stones (S) and detached urothelium, urothelial thickening, and a submucosal inflammatory infiltrate. Green dashed line demarcates the urothelial basement membrane. H&E, 20x objective. **(D)** Mixed inflammatory cellular infiltrate in a spontaneously infected *Mgb*-/- bladder urothelium and submucosa, with vacuolated cell remnants (inset) within the urothelium. Submucosal fibrosis is also evident. Trichrome, 20x objective, inset 40x. **(E)** A spontaneously infected *Mgb*-/- kidney demonstrates focal areas (yellow dashed lines) of intense interstitial leukocyte infiltration consisting of monocytes, lymphocytes, and plasma cells (inset). These areas are surrounded by normal-appearing renal parenchyma, consistent with a focal pyelonephritis. H&E, 5x objective; inset 40x objective. **(F)** In another spontaneously infected *Mgb-*/- kidney, the renal medulla demonstrates diffuse inflammatory infiltrate in the interstitium and suburothelial space. Medullary collecting ducts (*) are markedly dilated with cytoplasmic flattening, reactive nuclei, and luminal acute cellular degeneration consistent with acute tubular necrosis. Green dashed line demarcates the urothelial basement membrane. Trichrome, 20x objective.

We next examined the histology of bladders and kidneys of *Mgb-/-* mice with bladder stones. In the absence of bladder stones and bacteriuria, the *Mgb-/-* bladder exhibits distinct features compared to WT controls: detrusor hypoplasia, urothelial thickening, and luminal debris (Fig 3A and 3B), consistent with previous reports.[7,10] *Mgb*-/- bladders with stones displayed increased urothelial thickening, mucosal detachment, and histologic evidence of luminal stones (Fig 3C). A mononuclear and neutrophilic infiltrate occupied the urothelium and submucosa, with submucosal fibrosis evident on Trichrome stain (Fig 3D). Cytoplasmic vacuoles within the urothelium indicated acute cellular degeneration (Fig 3D inset). Infected *Mgb-/-* kidneys exhibited interstitial nephritis in the cortex and medulla (Fig 3E). Medullary collecting ducts were markedly dilated with cytoplasmic flattening, reactive nuclei, and luminal cellular degeneration consistent with acute tubular necrosis (Fig 3F). As in the bladder, trichrome staining identified collagen deposition immediately beneath the renal urothelium (Fig 3F). These features of cystitis or pyelonephritis observed in *Mgb-/-* urinary tracts with bladder stones were not evident in tissues from culture-negative *Mgb-/-*, *Mgb+/-* or WT mice (Fig 3A and 3B; data not shown).

### *Mgb-/-* hosts are susceptible to CONS experimental UTI

Based on the preponderance of CONS with urease activity among our stone bacterial isolates, along with the reported urease production by 55% of *Staphylococcus* species in humans with complicated UTIs [15], we reasoned that CONS was most likely responsible for bladder stones and UTI in *Mgb*-/- mice. We therefore investigated whether CONS isolated from spontaneously infected *Mgb*-/- mice could establish UTIs in uninfected *Mgb*-/- animals or WT controls. We isolated CONS strain 292 (CONS292) from a *Mgb-/-* bladder stone and confirmed urease activity. Next, after confirming urine sterility, we transurethrally injected 10^8^ colony forming units (CFU) of CONS292 into the bladders of 6 week old *Mgb*-/- or WT females. These mice were serially monitored for signs of morbidity as well as bacteriuria, crystalluria, urine pH and ultrasound changes.

**Fig 4:**
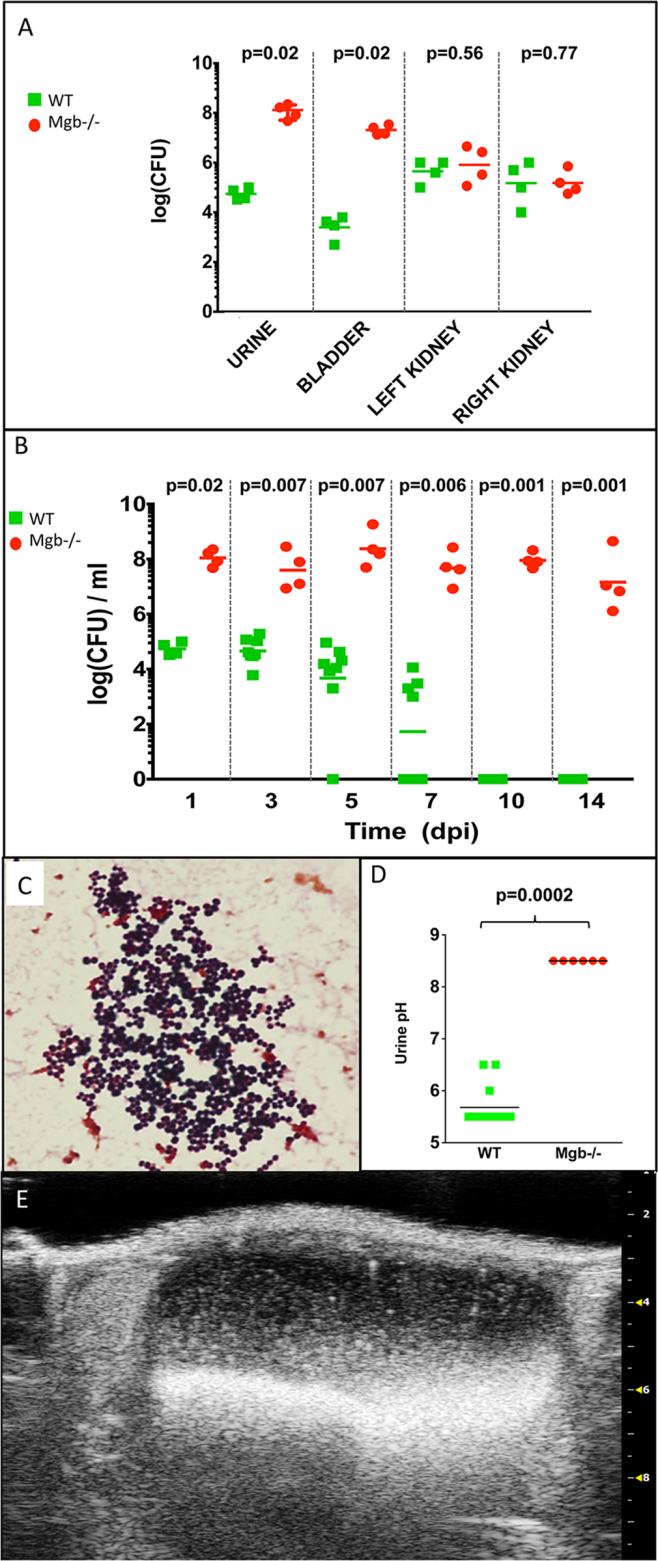
*Mgb*-/- mice develop persistent bacteriuria and alkaline pH following transurethral challenge with CONS292. (**A**) *Mgb*-/- mice exhibit increased magnitude of CONS292 recovery from urine and bladder than WT controls 1 dpi, but renal colonization is comparable. *p* values by Mann-Whitney U test are indicated above the graph. (**B**) Mgb-/- mice develop persistent bacteriuria, whereas WT control clear CONS292 from urine by 10 dpi. *p* values by Mann-Whitney U test are indicated above the graph. (**C**) Gram-positive cocci in clusters from *Mgb*-/- urine 14 dpi with 10^8^ CFU of CONS292. 100x objective. (**D**) *Mgb*-/- urine is uniquely alkaline compared to WT urine 10 dpi with CONS292. *p* value by Mann-Whitney U test is indicated above the graph. Similar results were obtained 3 dpi with p = 0.0001 (Data not shown). (**E**) Echogenic debris in a *Mgb-/-* bladder 14 dpi CONS292, confirmed as bladder stones at necropsy.

CONS292 initially colonized the urinary tracts of both *Mgb-/-* and WT mice (Fig 4A). However, we observed significantly less CONS burden in WT urine and bladders 1 day post infection (dpi), whereas renal colonization was comparable between genotypes (Fig 4A). CONS bacteriuria persisted for at least 2 weeks in *Mgb*-/- mice, whereas wild-type urine was sterile by 10 days post infection (dpi; Fig 4B). *Mgb*-/- urine uniquely contained Gram-positive cocci in clusters 14 dpi (Fig 4C). *Mgb*-/- urine pH was significantly more alkaline than WT urine as early as 3 dpi (p = 0.0001, data not shown) and at 10 dpi (p = 0.0002, Fig 4D). *Mgb-/-* mice infected with CONS292 demonstrated struvite crystalluria and bladder stones 14 dpi, whereas WT mice did not (Fig 4E).

**Fig 5:**
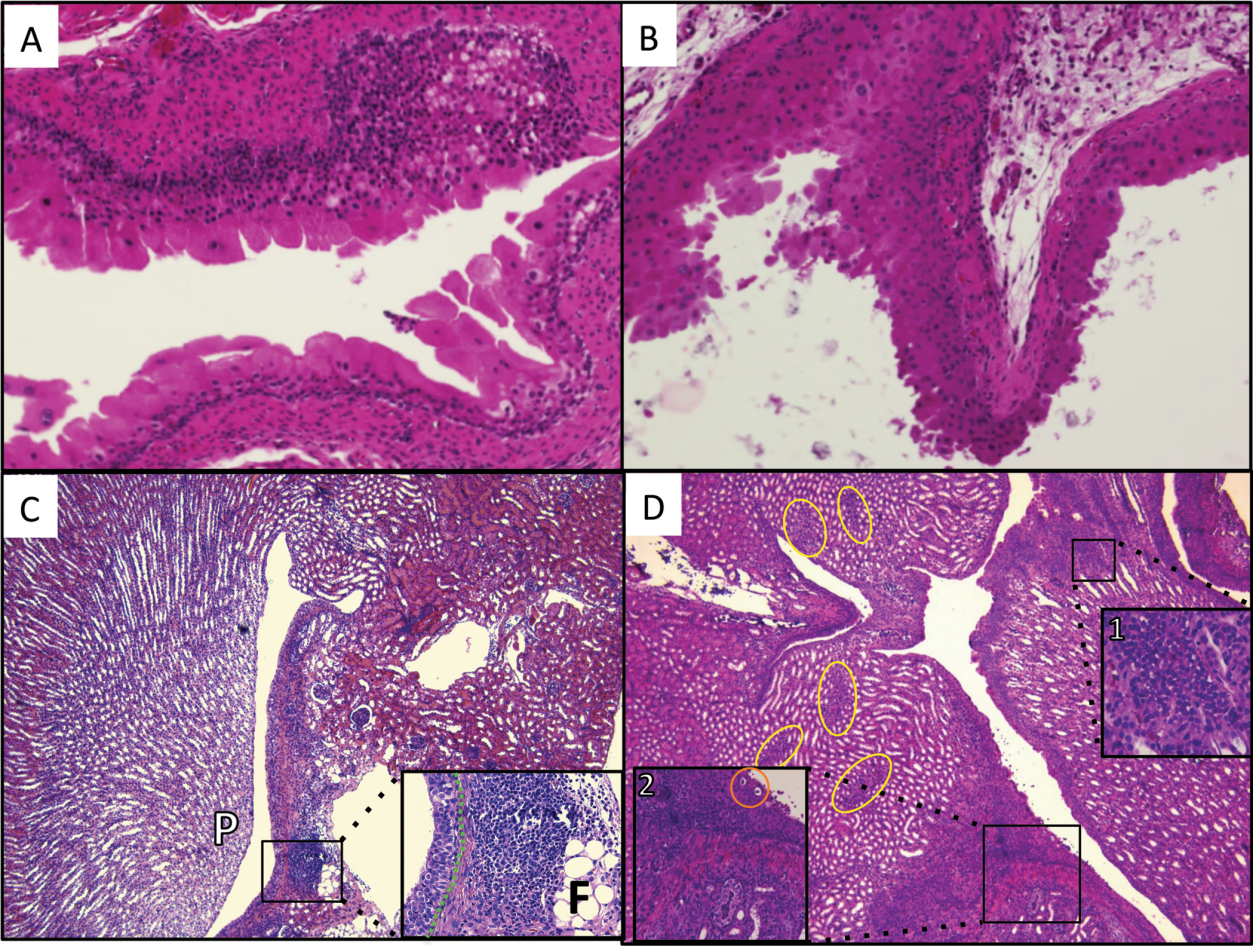
Experimental UTI with CONS292 recapitulates the spectrum of pathology observed in spontaneously infected *Mgb*-/- mice. **(A)** WT bladder exhibits regenerative apical urothelium and focal mononuclear submucosal infiltrate 14 dpi with CONS292. H&E, 20x objective **(B)** *Mgb*-/- bladder exhibits epithelial thickening, and desquamation with luminal crystals and submucosal edema 14 dpi with CONS292. H&E, 20x objective **(C)** Focal inflammatory cellular infiltrate in the urothelium and perihilar fat (F) was the only evidence of pathological changes in a WT kidney 14 dpi with CONS292. (P, renal papilla). Green dotted line (inset) demarcates the urothelial basement membrane. H&E, 5x objective; inset 20x objective **(D)** *Mgb*-/- kidney 14 dpi with CONS292 exhibits widespread changes including areas of dense mixed cellular infiltrate in the interstitium and tubules (inset1) as well as urothelial thickening, suburothelial inflammatory cells, and fibrosis (inset2). Nests of inflammatory cells are found scattered throughout the parenchyma (yellow ovals). Urothelial vacuolization is also present (orange circle, inset2). H&E, 5x objective; inset1 40x objective; inset2 20x objective.

Upon histological analysis 14 dpi, CONS-inoculated WT bladders exhibited prominent umbrella cells and a localized inflammatory infiltrate (Fig 5A). In contrast, CONS-infected *Mgb*-/- bladders demonstrated struvite crystals, urothelial thickening, and submucosal edema (Fig 5B). Inflammation in WT kidneys was absent in the renal parenchyma and restricted to the perihilar fat 14 dpi (Fig 5C). CONS-infected *Mgb*-/- kidneys manifested widespread interstitial and tubular inflammation with neutrophil-rich leukocyte casts in tubules and a vacuolated urothelium, as observed during spontaneous infection (Fig 5D).

### Urinary diversion reduces susceptibility of *Mgb-/-* mice to CONS experimental UTI

**Fig 6:**
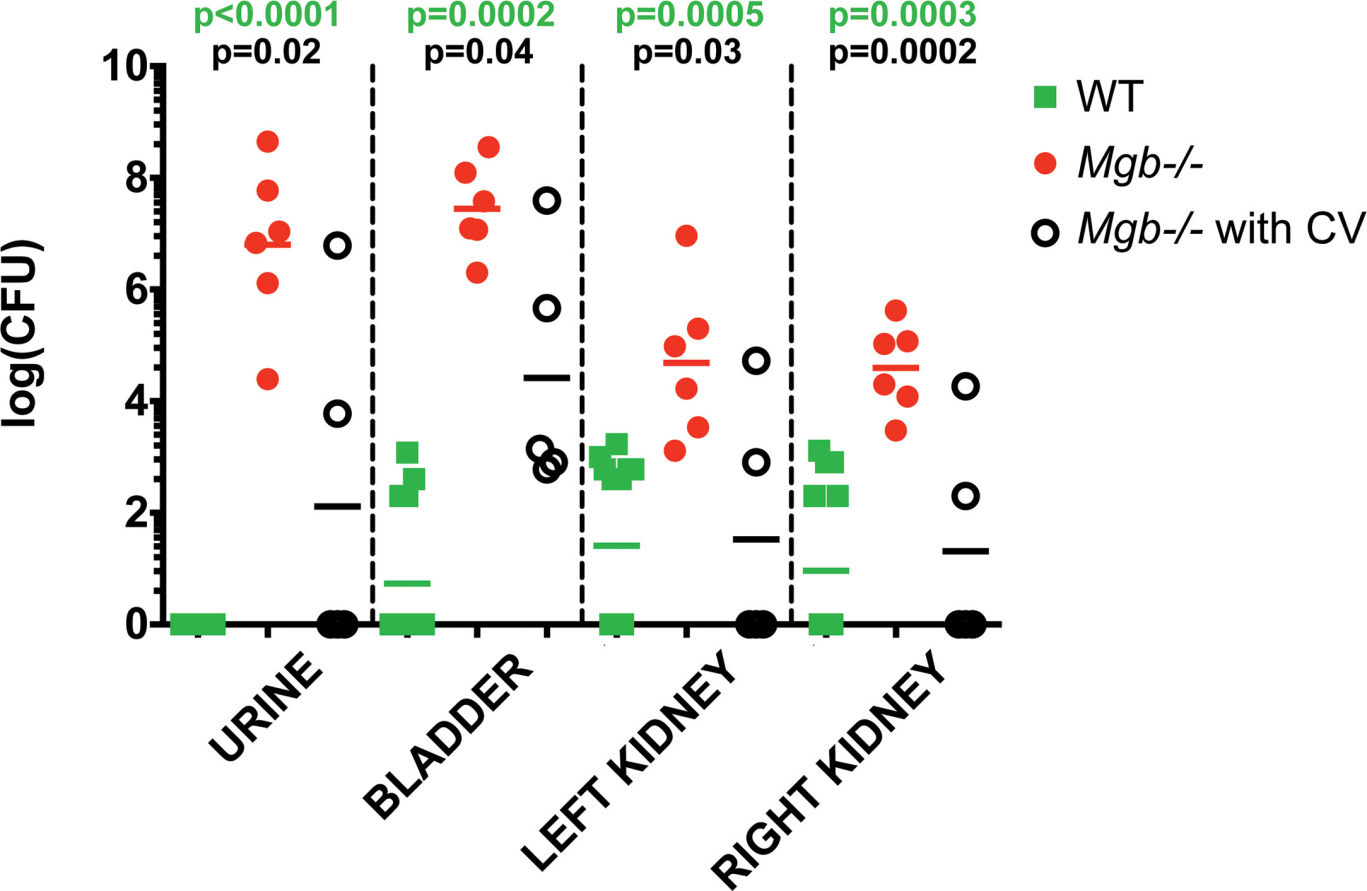
Urinary diversion decreases CONS292 susceptibility in *Mgb*-/- mice. *Mgb*-/- females post-CV (*Mgb-/-* CV) were transurethrally challenged with 10^8^ CFU of CONS292, along with undiverted *Mgb*-/- and WT mice. Bacteria were recovered 14 dpi from urine, bladder, and kidneys, then enumerated. *p* values by Mann-Whitney U test are indicated above the graph. A significantly higher magnitude of bacterial burden was observed in urine and all organs from undiverted *Mgb-/-* versus WT mice (green text) and versus *Mgb-/-* mice with CV (black text).

We hypothesized that the heightened susceptibility of the *Mgb*-/- urinary tract to CONS can be attributed at least in part to urine stasis. We therefore performed CV using absorbable polydioxanone suture in a cohort of uninfected *Mgb*-/- females, and confirmed the absence of urolithiasis, acidic pH, and sterility of the urine immediately prior to inoculation with 10^8^ CFU CONS292.[6,9,10] Following a 14 day period of observation, animals were euthanized and CONS extracted from urinary tracts to determine bacterial burden. As expected from our previous results, we recovered significantly more CONS from undiverted *Mgb*-/- than WT urine, bladders, and kidneys (Fig 6). WT bladders and kidneys consistently showed reduced bacterial burden relative to undiverted *Mgb*-/-, with 37.5% of WT organs demonstrating complete clearance of CONS compared to 0% in undiverted *Mgb*-/- mice. We also recovered significantly fewer CONS from urine, bladder, and kidneys of diverted *Mgb*-/- than from undiverted *Mgb*-/- animals. In the kidneys, bacterial burden was comparable between *Mgb*-/- with CV and WT (p = 0.79 [right kidney] and p = 0.88 [left kidney]). However, bladder (p = 0.0009) and urine (p = 0.02) CONS levels remained increased in *Mgb*-/- versus WT regardless of CV status (Fig 6).

## Discussion

COU is associated with increased risk and severity of UTI, and UTIs prolong hospitalization in patients with COU undergoing surgical repair.[2] The lack of animal models of spontaneous infection in the setting of COU has hampered the investigation of UTI risk factors in this patient population. We undertook this study in response to the anecdotal observation that *Mgb*-/- females develop spontaneous UTIs and struvite stones. *Mgb*-/- embryos demonstrate severely impaired detrusor muscle development, resulting in a highly compliant, atonic bladder in adults regardless of gender.[7] Adult *Mgb-/-* females dribble urine continuously, and gentle abdominal compression results in voids of one ml or more. *Mgb*-/- mice possess a large bladder volume, and the presence of urine at all times in the bladder argues that the animals never void to completion. Bladder ultrasound reveals nonspecific debris, as observed in human patients with urine stasis. The atonic, compliant *Mgb*-/- bladder parallels that found in patients with prune belly syndrome and non-neurogenic neurogenic bladder (Hinman syndrome). Such patients often develop complex UTIs, which are associated with unconventional uropathogens, prolonged hospitalization, and renal failure.[2,3,4] Spontaneous and experimental CONS UTI in *Mgb*-/- mice provides novel opportunities to model UTI and nephrolithiasis in the context of bladder dysfunction and urinary stasis.

In previous efforts to model UTI in patients with urinary tract obstruction, investigators have transurethrally administered human uropathogenic bacterial isolates to rodent models of acute acquired urinary tract obstruction, such as spinal cord transection, partial unilateral ureteral obstruction, or transient bladder outlet obstruction. These studies have shown that acutely obstructed urinary tracts exhibit increased magnitude and duration of bacterial burden and tissue injury following experimental UTI, compared to unobstructed units.[16,17,18,19] Rats with spinal cord injury exhibit exquisite susceptibility to UTI following transurethral administration of low (10^2^ CFU) bacterial inocula of uropathogenic *E*. *coli*, as well as increased bladder and renal inflammation.[16] In addition, acutely obstructed urinary tracts are uniquely susceptible to pyelonephritis following transurethral inoculation of bacterial strains that are non-pathogenic in unobstructed hosts, such as the laboratory K-12 *Escherichia* (*E*.) *coli* strain.[18,19]

Proposed mechanisms for heightened sensitivity of acutely obstructed urinary tracts to infection include urine stasis and incomplete bladder emptying; bacteremia as a consequence of bacterial vesicoureteral reflux at high pressures; and altered mucosal immune response to uropathogenic bacteria.[16,17,18,19] Spinal cord injury attenuates the induction of bladder transcripts with roles in the innate immune response typically seen following *E*. *coli* experimental UTI, such as *Il1b*, *Ltf*, *Cxcl3*, and *Tnf*, raising important questions about the regulation of gene expression by local bladder dynamics.[17] The chronicity of urine stasis, congenital inhibition of urine flow due to detrusor maldevelopment, and susceptibility to spontaneous CONS UTI are unique features that set the *Mgb-/-* model apart from existing models of experimental UTI in the setting of acute, postnatal urinary tract obstruction.

We identified CONS as the bacteria responsible for struvite stone formation and UTIs in this model system. This conclusion is supported by the role of urease in CONS pathogenicity [20]; isolation of CONS from stone cultures; visualization of Gram-positive cocci in clusters within stone matrix; and ability of CONS to generate struvite stones and UTIs in previously sterile urinary tracts of *Mgb*-/- mice following transurethral inoculation. CONS replicates slowly in urine compared to *Enterobacteriaceae*, so urine stasis likely accounts in part for the ability of CONS to accumulate in *Mgb*-/- urine.[21] Accordingly, *Mgb*-/- mice exhibit unique susceptibility to transurethral inoculation of CONS292, developing persistent bacteriuria and struvite urolithiasis compared to WT animals.

Urinary stasis in *Mgb*-/- may lead to increased luminal CONS and elevated local NH_4_^+^ concentrations via urease activity. The resulting alkaline pH favors precipitation of supersaturated calcium phosphate, together with magnesium.[15] While we previously identified non-absorbable suture as the nidus for struvite stones in male *Mgb-/-* mice following CV,[10] this study investigated spontaneous urolithiasis in *Mgb*-/- females without an antecedent surgery. The unique susceptibility of *Mgb*-/- hosts to CONS292 will enable studies to identify the nidus for stone formation in this setting, as well as to distinguish the roles of urease and other *Staphylococcus* virulence factors in UTI.[20,22,23]

Since we postulate a central role for urine stasis in the ability of CONS to act as a uropathogen, we expected that relief of stasis by cutaneous vesicostomy would reduce susceptibility of *Mgb*-/- animals to experimental UTI. We found that CONS-inoculated, vesicostomized *Mgb*-/- mice exhibited reduced renal bacterial burden, mirroring the clinical observation that urinary diversion protects the upper tract from infection. In contrast to the beneficial effect of CV on renal bacterial burden, bacterial burden in the *Mgb-/-* bladder remained significantly higher than WT controls despite CV. The impaired bladder clearance of CONS despite CV argues that local antimicrobial defenses are impaired in *Mgb*-/- bladders in a manner not entirely attributable to urinary stasis. Possible explanations for impaired bladder clearance of CONS in *Mgb*-/- following CV include: residual urinary stasis or altered bladder dynamics despite urinary tract decompression; increased bacterial attachment to urothelium; aberrant terminal differentiation of *Mgb*-/- urothelium leading to barrier disruption or epithelial dysfunction; impaired leukocyte recruitment; and/or poor production of antimicrobial peptides which rely upon regular bladder filling and emptying for their expression.

## Conclusions

*Mgb*-/- female mice exhibit susceptibility to spontaneous and experimental UTI, due at least in part to urinary stasis. Furthermore, CONS acts as an opportunistic murine uropathogen in the setting of stasis. The *Mgb-/-* model of spontaneous and experimental CONS infection complements studies of superimposed infection in acquired obstructed uropathy such as spinal cord injury, transient bladder obstruction, and partial ureteral obstruction, allowing the dissection of host and bacterial factors that govern infection susceptibility.
